# The double-edged role and therapeutic potential of TREM2 in atherosclerosis

**DOI:** 10.1186/s40364-024-00675-w

**Published:** 2024-11-04

**Authors:** Botao Zhu, Yuxuan Liu, Daoquan Peng

**Affiliations:** Department of Cardiovascular Medicine, The Second Xiangya Hospital of Central South University, No.139 Middle Renmin Road, Changsha, Hunan 410011 China

**Keywords:** TREM2, Atherosclerosis, Plaque stability, Macrophage, Therapeutic strategies

## Abstract

Atherosclerosis is a chronic lipid-driven inflammatory disease characterized by infiltration of large numbers of macrophages. The progression of the disease is closely related to the status of macrophages in atherosclerotic plaques. Recent advances in plaque analysis have revealed a subpopulation of macrophages that express high levels of triggering receptor expressed on myeloid cells 2 (TREM2). Although TREM2 is known to play a critical role in inflammation, lipid metabolism, and tissue repair, its role in atherosclerosis is still not fully understood. Recent studies have shown that TREM2 promotes macrophage cholesterol uptake and efflux, enhances efferocytosis function, regulates inflammation and metabolism, and promotes cell survival, all of which are significant functions in atherosclerosis. In early plaques TREM2 promotes lipid uptake and increases lesion size. In advanced plaques TREM2 promotes macrophage survival and increases plaque stability. The dualistic nature of TREM2 in atherosclerosis, where it can exert both protective effect and a side effect of increased lesion size, presents a complex but crucial area of study. Understanding these dual roles could help in the development of new therapeutic strategies to modulate TREM2 activity and utilize its atheroprotective function while mitigating its deleterious effects. In this review, we discuss the roles and mechanisms of TREM2 during different stages of atherosclerotic plaques, as well as the potential applications of TREM2 in the diagnosis and treatment of atherosclerosis.

## Introduction

More than 17 million people worldwide die from cardiovascular diseases (CVD), with ischemic heart disease and stroke being the primary culprits behind the global CVD burden (49% and 35% of the total burden, respectively) [1]. Atherosclerotic cardiovascular disease remains the predominant cause of CVD globally [2, 3]. Atherosclerosis is a chronic inflammatory disease initiated by the accumulation of apolipoprotein B-containing lipoproteins such as low-density lipoproteins (LDL) in the arterial intima. Macrophages internalize lipoproteins deposited in the arterial wall and become foam cells. The retention of foam cells within atherosclerotic plaques, coupled with unresolved inflammation, contributes to the progression of atherosclerosis and the destabilization of plaques [4, 5]. Excessive LDL uptake induces macrophage apoptosis and is cleared through a process called efferocytosis, which causes secondary necrosis when this process is limited [6]. Some lesions subsequently form a necrotic core, impeding blood flow and triggering tissue ischemia, which can lead to severe clinical consequences such as ischemic heart disease and ischemic strokes [7]. Therefore, it is imperative to gain a deeper comprehension of its underlying causes and explore therapeutic strategies for alleviating the burden of atherosclerosis.

Triggering receptor expressed on myeloid cells 2 (TREM2), an immunomodulatory receptor, has been the subject of extensive research in both the nervous and immune systems. In recent years, it has been found to be associated with neurodegenerative diseases, particularly Alzheimer’s disease (AD) [8–11]. Research on TREM2 within the central nervous system has revealed its role as a lipid receptor responsible for regulating cholesterol and phospholipid metabolism [12, 13]. TREM2 also plays an essential part in peripheral lipid metabolism [14–16]. TREM2 appears to be active in lipid-rich pathological environments. Moreover, the roles of TREM2 have been found to include activation and maintenance of immune cell function, modulation of inflammation, and tissue repair and protection [17–22]. With the aid of single-cell RNA sequencing, macrophages with high expression of TREM2 (TREM2hi) were detected in plaques at different stages of atherosclerosis in mice [23, 24]. TREM2hi macrophages appear to possess distinct functions related to lipid metabolism and efferocytosis, which are involved in atherosclerosis progression. Although the relationship between TREM2 and atherosclerosis is still in the early stages of research, some exciting findings have emerged. Building on current understanding, we can also draw insights from the role of TREM2 in other diseases to explore its potential impact and therapeutic applications in atherosclerosis.

In this review, we summarize the latest research developments on TREM2 and its role and mechanisms in atherosclerosis. Additionally, we discuss potential diagnostic and therapeutic approaches for atherosclerosis that target TREM2.

## Structure and function of TREM2

**Fig. 1:**
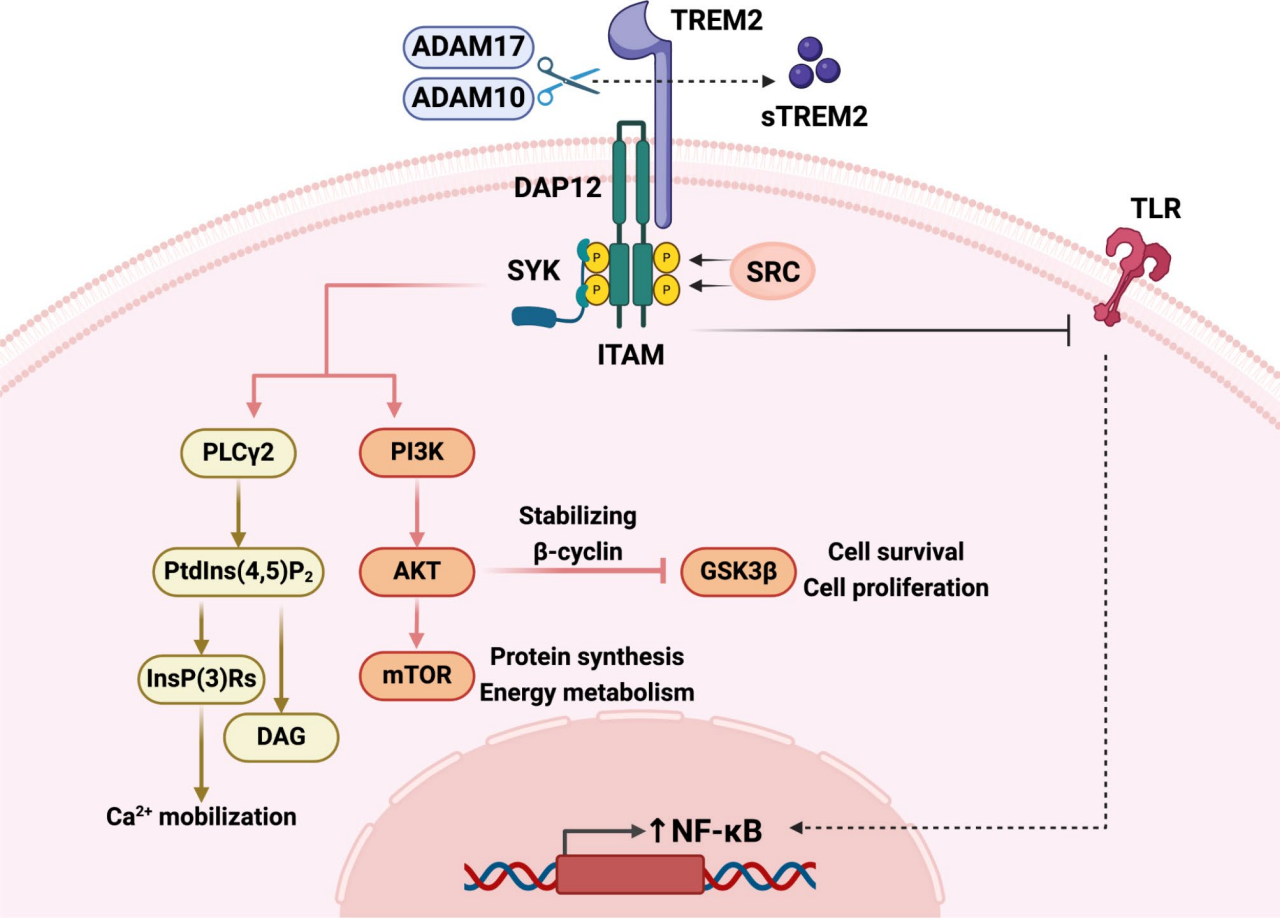
Structural features and basic functions of triggering receptor expressed on myeloid cells 2 (TREM2). TREM2 consists of an extracellular domain, a separate transmembrane domain, and a short cytoplasmic domain that does not have any signaling function. TREM2 binds to the DNAX activating protein 12 (DAP12) protein via the transmembrane domain. Triggered by TREM2, the activation motif of the immunoreceptor tyrosine (ITAM) in the cytoplasmic domain of DAP12 is phosphorylated by members of the SRC kinase family. The phosphorylated ITAM recruits the protein tyrosine kinase (SYK) to activate phosphatidylinositol 3-kinase (PI3K) and phospholipase Cγ2 (PLCγ2). Activated by PI3K, the kinase AKT supports protein synthesis and energy metabolism by activating the mechanistic target of rapamycin (mTOR) pathway. AKT can inactivate glycogen synthase kinase 3β (GSK3β) by stabilizing β-cyclin, contributing to cell survival and proliferation. PLCγ2 catalyzes the degradation of phosphatidylinositol 4,5-bisphosphate (Ptdlns(4,5)P_2_) to inositol 1,4,5-trisphosphate (InsP(3)Rs) and diacylglycerol (DAG). InsP(3)Rs induces intracellular Ca^2+^ mobilization. TREM2 can also inhibit the activation of Toll-like receptors (TLRs), which are capable of activating NF-κB. The α-secretases disintegrin and metalloproteinase-17 (ADAM17) and ADAM10 can cleave the extracellular domain of TREM2 at the H157-S158 peptide bond, which releases soluble TREM2 (sTREM2)

TREMs are a family of cell-surface receptors widely expressed on myeloid cells. Human TREM gene clusters are located on human chromosome 6p21.1 [17]. TREM2 consists of three structural domains: an extracellular domain with a single V-type immunoglobulin domain, a separate transmembrane domain, and a short cytoplasmic domain that does not have any signaling function [17]. Due to the lack of a prominent signaling motif in the intracellular domain, TREM2 binds to adaptor proteins such as DNAX activating protein 12 (DAP12) and DAP10 via positively charged residues in the transmembrane domain [25, 26]. Triggered by TREM2, the activation motif of the immunoreceptor tyrosine (ITAM) in the cytoplasmic domain of DAP12 is phosphorylated by members of the SRC kinase family. The phosphorylated ITAM recruits spleen tyrosine kinase to activate downstream signaling molecules such as phosphatidylinositol 3-kinase (PI3K) and phospholipase Cγ2 (PLCγ2). Activated by PI3K, the kinase AKT supports protein synthesis and energy metabolism by activating the mechanistic target of rapamycin (mTOR) pathway. Additionally, AKT can inactivate glycogen synthase kinase 3β (GSK3β) by stabilizing β-cyclin, contributing to cell survival and proliferation [27]. As for PLCγ2, it catalyzes the degradation of phosphatidylinositol 4,5-bisphosphate to inositol 1,4,5-trisphosphate and diacylglycerol. 1,4,5-Trisphosphate induces intracellular Ca^2+^ mobilization [17, 28]. Moreover, TREM2 can inhibit the activation of Toll-like receptors (TLRs), which are capable of activating nuclear factor kappa B (NF-κB) [17](Fig. 1).

Notably, the signaling pathways mediated by TREM2 and their functions may differ in various cell types or under different physiological or pathological conditions [18]. Previous studies found that in the basal state, TREM2 maintains myeloid cell survival and maturation [29–31], regulates inflammation [32], and ensures that myeloid cells remain ready and capable of responding to injury and disease. In addition, TREM2 can enhance phagocytosis or act as a negative regulator of inflammation to drive myeloid cells to respond to stimuli under conditions of injury or disease [33, 34]. Overall, TREM2 performs multiple functions, mainly through DAP12 and DAP10 signaling integrated with other signaling pathways. However, the downstream mechanisms of TREM2 activation may be more complex and need further research to be better understood.

The extracellular domain of TREM2 can bind to a large number of different ligands, such as cells, bacterial anionic molecules, lipoproteins, and nucleic acid. It is worth noting that the α-secretases disintegrin and metalloproteinase-17 (ADAM17) and ADAM10 can cleave the extracellular domain of TREM2 at the H157-S158 peptide bond, which releases soluble TREM2 (sTREM2) [35–38](Fig. 1). The function and significance of TREM2 shedding are still being studied. In a transgenic mouse model with reduced TREM2 shedding, increasing TREM2 receptor loading on myeloid cells improved cell viability and function [39]. On the other hand, an increase in extracellular domain shedding led to a reduction in the number of full-length TREM2 on the cell surface and decreased TREM2-dependent phagocytosis [36]. sTREM2 can serve as a biomarker detected in serum and cerebrospinal fluid [22, 40–42]. In addition to that, sTREM2 may have a biological function. It is believed that sTREM2 can inhibit further TREM2 activation by binding to TREM2 ligands [17]. In AD, sTREM2 can inhibit the secondary nucleation of amyloid-β (Aβ) fibrillization and enhance the cellular uptake of fibrillar Aβ [43]. On the other hand, sTREM2 might have pro-survival effects on cells. Specifically, sTREM2 induced an inflammatory response in microglia and promotes their survival [44]. There is also evidence suggesting that sTREM2 can prevent macrophage apoptosis following respiratory viral infections [45]. Interestingly, in vivo, injection of sTREM2 has significantly enhanced the function and structure of infarcted hearts [46].These multifaceted roles of sTREM2 highlight its importance in a variety of pathological situations.

In conclusion, TREM2 is known to be involved in various metabolic and inflammatory diseases associated with myeloid cells. Atherosclerosis is one of such diseases where TREM2hi macrophages play a significant role, impacting several immune and metabolic processes. More research is needed to determine whether TREM2 functions similarly in atherosclerosis, which can help identify pathological mechanisms and therapeutic strategies for this disease.

## The role and mechanism of TREM2 in atherosclerosis

### TREM2hi macrophages in atherosclerosis

Atherosclerosis is a progressive inflammatory disease of the arterial wall characterized by extensive infiltration of macrophages, which play both beneficial and detrimental roles [47]. Traditionally, macrophages in atherosclerosis have been studied using a simple M1/M2 polarization model, which has limitations as it does not reflect the natural state of macrophages in vivo [48]. However, a more comprehensive understanding of the different cell types and their phenotypes in atherosclerotic plaques is now possible because of recent advancements in single-cell technology [49, 50]. In mouse models of atherosclerosis, three main subpopulations of macrophages have been identified, including resident-like, pro-inflammatory, and anti-inflammatory foamy TREM2hi macrophages [51]. Gene ontology term analyses revealed that a distinct subpopulation of *Trem2*^*+*^*Cd9*^*+*^ macrophages in the artery was enriched with specific functions not observed in other macrophage populations, such as lipid metabolic processes, regulation of cholesterol efflux, and oxidative stress [50]. Moreover, immunostaining showed that *Cd9*^*+*^ macrophages appeared to preferentially localize within and around necrotic areas, suggesting that they may play a critical role in plaque stability [50]. These macrophages have been identified as TREM2hi macrophages, which express increased levels of various genes such as *Trem2*, *Cd9*, secreted phosphoprotein 1 (*Spp1*), hydrogen voltage-gated channel 1 (*Hvcn1*), galectin-3 (*Lgals3*), and Cathepsin B (*Ctsb*) [49, 51–54]. CD9 is predominantly considered an anti-inflammatory marker in macrophages and is associated with CD36-mediated foam cell formation [55, 56]. Galectin-3 acts as a TREM2 upstream factor to regulate macrophage polarization and is also a biomarker of atherosclerosis [57, 58]. Cathepsin, a lysosomal protein hydrolase in macrophages, promotes inflammation and reduces plaque stability in atherosclerosis [58, 59]. Pathway analysis of TREM2hi macrophages revealed an association with intracellular lipid accumulation and foam cell formation, with enrichment in lipid metabolism, cholesterol efflux regulation, and lysosome-related functions [50, 52, 60].

It has been discovered that similar macrophage subpopulations exist in human atherosclerotic plaques. Researchers recently analyzed scRNA-seq data from humans and mice and reported that markers indicating macrophage subpopulations were consistently distributed in atherosclerotic lesions in both species [49, 61]. The gene expression patterns within the three main human macrophage subpopulations (hInflammatory macrophages, hFoamy macrophages, and hLYVE1 macrophages) were comparable to those of the mouse macrophage subpopulations (pro-inflammatory macrophages, foamy/TREM2hi macrophages, and resident macrophages), respectively [49]. According to Gene Ontology analysis, hFoamy macrophages were enriched in functions related to lipid metabolism [49]. Moreover, it has been observed that disease-associated microglia (DAMs) [62], lipid-associated macrophages (LAMs) [16], and nonalcoholic steatohepatitis-associated macrophages (NAMs) [63] exhibit a cellular transcriptional state similar to that of TREM2hi macrophages. These macrophages share the commonality of being in a lipid-rich and chronically inflamed environment. Therefore, the development of the TREM2hi macrophage state may be closely associated with lipid metabolism and loading.

However, unlike the mouse model of atherosclerosis, which is relatively homogeneous, patients present a highly diverse group due to factors such as age, gender, and comorbidities such as diabetes. These elements influence the nature of immune cells in atherosclerotic plaques [64–66]. Therefore, it is necessary to expand the sample size in future studies to minimize the interference of individual heterogeneity on the analysis results.

### TREM2 increases lesion size in early plaques

**Fig. 2:**
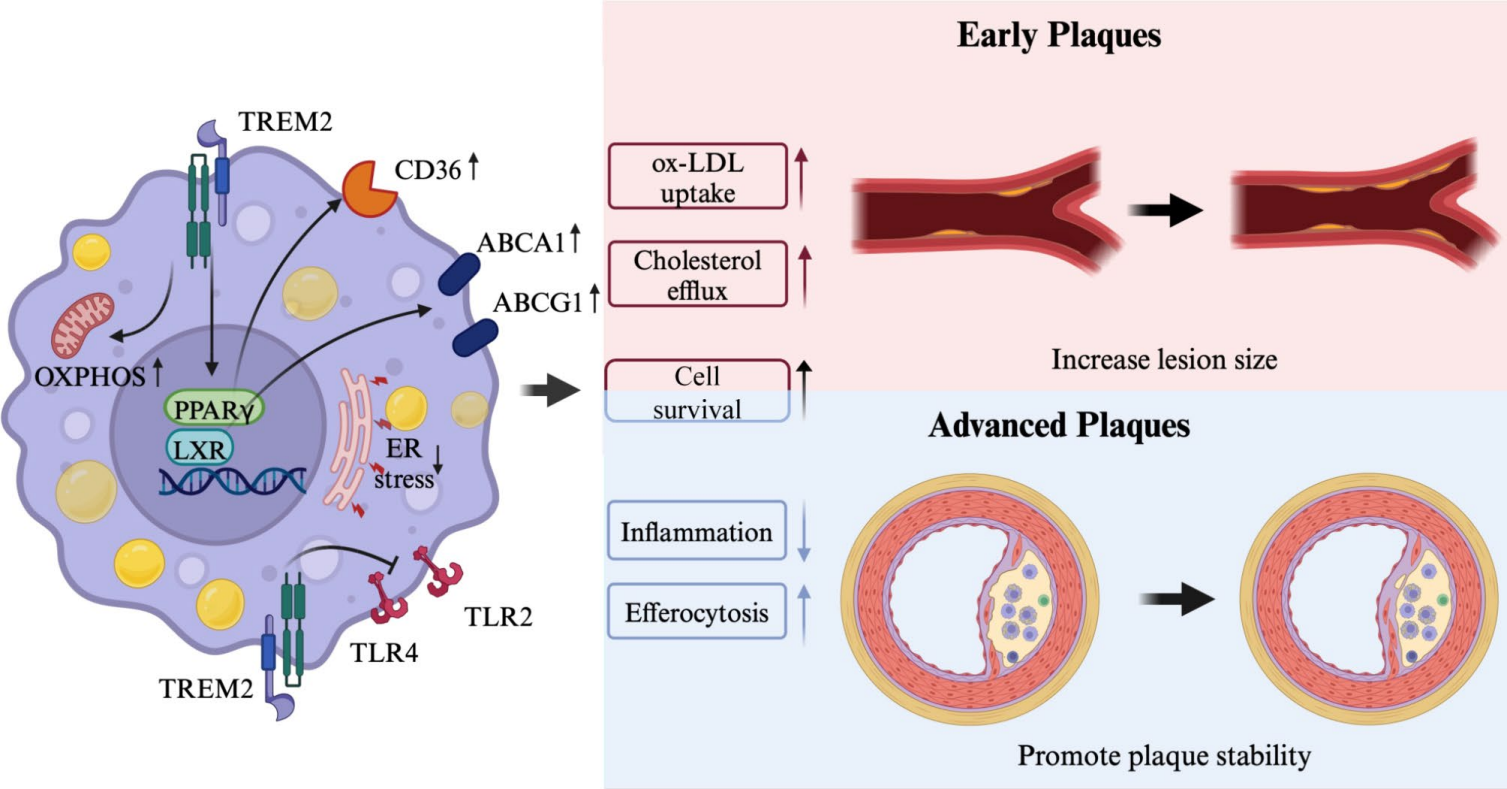
The role and mechanism of triggering receptor expressed on myeloid cells 2 (TREM2) in atherosclerosis. In early plaques, TREM2 increases lesion size because TREM2 promotes the expression of CD36, which is associated with lipid uptake, by increasing peroxisome proliferator-activated receptor gamma (PPARγ) activation. Additionally, TREM2 promotes cholesterol efflux by activating liver X receptor (LXR) and upregulating the expression of ATP-binding cassette transporter A1 (ABCA1) and ATP-binding cassette transporter G1 (ABCG1), which reduces endoplasmic reticulum (ER) stress and helps to maintain the survival of cells. The increased survival of macrophages results in relatively greater uptake of lipids. However, in advanced atherosclerotic plaques, TREM2 plays a protective role by enhancing macrophage efferocytosis function and modulating inflammation, which reduces the risk of plaque necrotic core formation and rupture. TREM2 can inhibit Toll-like receptor (TLR) induced amplification of inflammatory signals. When treated with TREM2 agonists, the metabolism of foamy macrophages was reprogrammed to oxidative phosphorylation (OXPHOS), promoting cell survival. Macrophage survival in advanced plaques would contribute to plaque stabilization. ox-LDL indicates oxidized low-density lipoprotein

Current research indicates that in the early stages of atherosclerosis, TREM2 promotes lipid uptake by macrophages in response to arterial wall lipid loading [67–70]. Additionally, TREM2 enhances cholesterol efflux in macrophages, which appears to be a compensatory mechanism to manage excess lipids by maintaining macrophage survival [68, 69]. However, this mechanism may also have a side effect—exacerbating lesion expansion [67–69] (Fig. 2). The following sections discuss the specific mechanisms involved.

#### TREM2 promotes lipid uptake in macrophages

Dysfunction in lipid metabolism within macrophages is a significant cause of the progression of atherosclerotic plaques. TREM2 may affect atherosclerosis by regulating macrophage lipid absorption and processing. In mice, TREM2 expression increased over time during atherosclerotic plaque formation [67]. TREM2 is essential for foam cell formation in atherosclerotic plaques. In vitro CRISPR screening identified TREM2 as a key gene regulating oxidized low-density lipoprotein (oxLDL) uptake by macrophages [68]. By differentiating BV2 myeloid cells into foamy macrophages and using fluorescence-activated cell sorting technology to sort cells containing DiI-oxLDL, the researchers found that TREM2 significantly enhanced oxLDL uptake and was the most enriched gene associated with oxLDL uptake [68]. Aortic atherosclerotic lesion size was significantly reduced in TREM2 conditional knockout mice and was not associated with changes in cholesterol or body weight [68]. Lipid uptake efficiency was significantly decreased in *Trem2*^*−/−*^ macrophages compared to control, whereas treatment with the TREM2 agonist increased the uptake [68, 70]. Differential expression analysis of *Trem2*^*−/−*^ foam macrophages revealed decreased expression of foam macrophage markers *Spp1* and *Cd5l*, lipid uptake-related genes *Msr1* and *Cd36*, and the survival-related gene *Bcl2* [70]. In addition, macrophages overexpressing TREM2 showed a significant increase in lipid uptake, which may be attributed to the ability of TREM2 to increase the expression of CD36 by inhibiting the phosphorylation level of the transcription factor peroxisome proliferator-activated receptor gamma [67]. CD36 plays an essential role in the uptake of oxidized LDL and the activation of downstream inflammatory responses [71]. Consistently, primary microglia obtained from TREM2 deficient mice showed a 40% reduction in LDL uptake compared to wild-type mice in AD [72]. These results demonstrated the involvement of TREM2 in upregulating cellular lipid uptake, which has a significant impact on the atherosclerotic process.

#### TREM2 promotes cholesterol efflux to maintain macrophage survival

Macrophage death in atherosclerotic plaques is usually associated with cholesterol overload because excess free cholesterol causes membrane damage [73]. Another factor contributing to slowed lipid uptake in TREM2-deficient macrophages may be decreased cholesterol efflux and impaired cell survival. The activation of liver X receptor (LXR) in macrophages induces cholesterol efflux by upregulating ATP-binding cassette transporter A1 (ABCA1) and ATP-binding cassette transporter G1 (ABCG1) [74, 75]. Downregulation of the expression of the cholesterol efflux genes *Abca1* and *Abcg1* occurred in TREM2-deficient foam cells, resulting in cholesterol accumulation and subsequent cell death [68]. Patterson et al. demonstrated that treating *Ldlr*^*−/−*^ mice with AL002a led to an increase in plaque size, which was associated with enhanced cholesterol efflux capacity of macrophages driven by TREM2 within the lesions [69]. The inability of TREM2-deficient macrophages to adapt to excess cholesterol exposure is likely to be associated with endoplasmic reticulum (ER) stress. ER stress inhibitors restored TREM2-deficient macrophage viability to wild type levels [68]. Notably, treatment with LXR agonists also increased cell survival because LXR activation promoted cholesterol efflux to reduce ER stress [68, 76]. It has been demonstrated that the deficiency of TREM2 in microglia leads to the accumulation of cholesteryl esters within the cells [13]. However, treatment with LXR agonists can rescue this phenotype by increasing gene expression in cholesterol efflux and promoting phagocytic processes [13]. In a study of atherosclerotic plaques from a myeloid LXR-deficient mouse model, RNA analysis revealed that LXR-deficient foamy macrophages presented relatively low TREM2 expression [77]. At the same time, the expression of endocytosis and lipid metabolism genes was downregulated, while the expression of inflammatory genes was significantly upregulated in these cells [77]. Reduced cellular cholesterol efflux may exacerbate the inflammatory response by the activation of NLRP3 inflammasome [78]. Collectively, these results suggested that TREM2 may enhance cholesterol efflux by activating LXR to maintain cell survival so that macrophage lipid uptake would not be slowed by cell death, which contributes to the increase in lesion size.

### TREM2 promotes plaque stability in advanced plaques

Despite the potential for lesion enlargement, the significance of TREM2 in stabilizing advanced atherosclerotic plaques should not be overlooked. It promotes macrophage efferocytosis to remove apoptotic cells and regulates the level of inflammation and metabolic adaptation within the plaque [68–70]. In cases where plaque has already formed, maintaining plaque stability by reducing the necrotic core is prognostically beneficial in atherosclerosis [69, 70] (Fig. 2). The following sections discuss the specific mechanisms involved.

#### TREM2 maintains macrophage efferocytosis

TREM2 maintains plaque homeostasis by participating in efferocytosis processes. Impaired macrophage efferocytosis is a feature of unstable plaques, which indicates unresolved inflammation and larger necrotic cores in the plaque [73]. In the lesions of mice with *Trem2*^*−/−*^ bone marrow, macrophage efferocytosis activity is reduced [70]. Single-nucleus RNA-sequencing and in vitro experiments demonstrated that TREM2 enhances macrophage efferocytosis, as *Trem2*^*−/−*^ macrophages exhibited reduced ability to clear apoptotic cells [70]. The activation of TREM2 enhanced the expression of genes such as *Mertk* and *Il10*, which were essential for efferocytosis and inflammation resolution, whereas this response was blunted in *Trem2*^*−/−*^ bone marrow-derived macrophages (BMDMs) [70]. Furthermore, 2-step efferocytosis assay demonstrated that TREM2 deficiency also impairs the continuous efferocytosis ability of macrophages [70]. The association between TREM2 deficiency and impaired efferocytosis is evident in both foam and non-foam macrophages [68]. Impaired efferocytosis function leads to secondary necrosis and stimulation of inflammatory cytokine release, increasing the risk of plaque rupture. In nonalcoholic steatohepatitis (NASH), TREM2 promoted the ability of macrophages to clear apoptotic cells, limiting the deterioration of NASH [79, 80]. A similar phenotype was observed in DAM [81]. Due to the similarity in gene expressions with DAM, LAM, and NAM [60, 82], atherosclerosis-associated TREM2hi macrophages are expected to be involved in atherosclerosis through a similar mechanism. Thus, more experiments are needed to explore the mechanisms of how TREM2 promotes and maintains macrophage efferocytosis in atherosclerotic plaques.

#### TREM2 regulates macrophage inflammation

Persistent inflammation is a critical driver of plaque necrosis, promoting the progression and severity of atherosclerotic lesions [6]. On the one hand, TREM2 can promote the efferocytosis activity of macrophages to clear accumulated necrotic debris in plaques, which can otherwise exacerbate plaque inflammation. On the other hand, TREM2 influences the progression of atherosclerosis by modulating inflammatory pathways in macrophages. TREM2 agonist AL002a-treated atherosclerotic mice presented a reduction in necrotic core area of approximately 1/4 [69]. Pathway analysis indicated that AL002a treatment downregulated inflammatory and apoptotic pathways, suggesting that TREM2 agonism promotes cell survival and attenuates inflammation [69]. This transcriptional profile aligns with features of human asymptomatic carotid artery plaques [69]. In human atherosclerotic plaques, mainly non-foam macrophages play a pro-inflammatory role. Foam macrophages with high TREM2 expression mainly express genes related to lipid processing and expressed significantly fewer inflammatory cytokines such as interleukin (IL)-1β [52]. TLR signaling, such as TLR2 and TLR4, has well-defined roles in atherosclerotic plaque formation which promotes macrophage inflammatory phenotypes and cholesterol deposition [83]. TREM2 can inhibit TLR-induced amplification of inflammatory signals by reducing cytokine production [32, 84]. On the other hand, TREM2-deficient NAM secreted more pro-inflammatory cytokines, such as TNFα, IL-1β, and IL6, which exacerbated the pro-inflammatory response induced by triglyceride-rich lipoproteins [79]. Meanwhile, TNF and IL-1β can induce TREM2 protein shedding through the activation of ADAM17, causing a vicious cycle [80].TREM2 regulates the inflammatory response of immune cells to maintain a moderate level. Excessive inflammatory response is closely associated with the deterioration of atherosclerosis, so the role of TREM2 in this process may be very important.

#### TREM2 reprograms the metabolism of macrophages to maintain macrophage survival

Increased intraplaque cell death is an important factor in plaque destabilization and rupture [47]. *Trem2*^*−/−*^ BMDMs showed lower survival under cholesterol-loading conditions [70]. This suggested that TREM2 plays a role in promoting the survival of foamy macrophages. BV2 cells and peritoneal macrophages lacking TREM2 also showed notably impaired survival under lipid-loaded conditions [68]. Researchers have suggested that the lack of the oxidative phosphorylation (OXPHOS) pathway could be responsible for the imbalance in cellular homeostasis and increased death of macrophages when processing lipids [85]. In macrophages treated with the TREM2 agonist AL002a, there was an increased expression of lysosomal and OXPHOS genes, as well as genes involved in collagen production [69]. The impact of TREM2 agonism on OXPHOS was investigated using a mitochondrial stress test in non-foamy and foamy BMDMs pretreated with isotype or AL002a antibody. Non-foamy macrophages showed minimal differences in oxygen consumption rates, whereas foamy macrophages treated with AL002a exhibited increased basal and ATP-linked oxygen consumption rates, indicating elevated OXPHOS in these cells [69]. Elevated mitochondrial potential in TREM2 agonist-treated BMDMs was observed in another study [70]. Additionally, AL002a treatment has been found to reprogram vascular smooth muscle cells (VSMCs)-derived foamy cells, leading to increased collagen production and extracellular matrix organization [69]. Interestingly, although TREM2 is predominantly expressed in mononuclear phagocytes, relatively low levels of TREM2 expression have also been detected in VSMCs [69, 70]. Guo et al. suggested that TREM2 facilitates lipid uptake in VSMCs [67]. This suggests that targeting TREM2 could potentially influence cells other than macrophages. It has been suggested that TREM2 signaling in macrophages may affect other arterial cell subpopulations, possibly by enhancing TGFβ signaling [69]. Further studies using cell type-specific models are needed to reveal the roles of TREM2 in non-macrophage cells within atherosclerotic plaques.

### TREM2 and systemic lipid level

There is evidence that TREM2 and its downstream signaling are important pathways for maintaining lipid homeostasis. In TREM2-deficient mice and mice transplanted with *Trem2*^*−/−*^ bone marrow, the recruitment of LAM to hypertrophied adipocytes is hindered, and the formation of crown-like structures around the dead adipocytes is incapable [15, 16]. This ultimately leads to adipocyte hypertrophy, insulin resistance, and hypercholesterolemia. Elevated LDL cholesterol levels are a significant risk factor for atherosclerosis. Thus, from this perspective, TREM2 in areas other than atherosclerotic plaques, such as adipose tissue, can affect atherosclerosis by influencing systemic lipid levels. However, whether TREM2 affects systemic lipid levels in the context of atherosclerosis remains to be investigated. In the study by Piollet et al., transplantation of *Trem2*^*−/−*^ bone marrow did not significantly affect the body weight or systemic lipid levels in mice under atherosclerotic conditions [70]. Similarly, another study found that treatment with a TREM2 agonist did not alter the body weight or serum cholesterol levels in *Ldlr*^*−/−*^ mice [69]. Additionally, Patterson et al. reported that the deletion of *Trem2* in macrophages did not result in systemic cholesterol effects [68]. Nevertheless, given the possibility that global TREM2 knockout might elevate systemic cholesterol levels, future studies on the role of TREM2 in atherosclerosis require more precise mouse models, such as conditional knockout or bone marrow reconstitution. In addition, the use of *Apoe*^*−/−*^ mice may interfere with studies on TREM2, as APOE is a known downstream effector of TREM2 [86].Here, we summarize the mouse models used in current atherosclerosis research and their respective findings for reference (Table 1).

**Table 1:** Mouse models used in current atherosclerosis research and their respective findings

Models	TREM2 level	Lesion size	Necrotic core	Systematic lipid level	Macrophages studied	Macrophages phenotypes	Ref.
Atherogenic *Cx3cr1-CreERT2-Trem2*^*flox*^ mice	↓	↓	=	=	Macrophages in the aorta	Decreased cell survival	[68]
					BV2 cells; peritoneal macrophages	Deficiency in cholesterol efflux and decreased cell survival	
AL002a (Trem2 agonist) treated atherogenic *Ldlr*^*−/−*^ mice	↑	↑	↓	=	*Trem2*^*+*^*Lgals3*^*+*^*Fabp5*^*+*^macrophages from the aorta	Increased oxidative phosphorylation and decreased inflammation	[69]
					BMDMs	Increased cholesterol efflux, oxidative phosphorylation, and cell survival	
Atherogenic *ApoE*^*−/−*^*Trem2*^*−/−*^mice	↓	↓	↓	=	BMDMs; Raw264.7 cells	Decreased lipid intake	[67]
Atherogenic *Ldlr*^*−/−*^*Trem2*^*−/−*^ mice	↓	=	↑	=	*Gpnmb*^*+*^foamy macrophages from the aorta	Decreased lipid intake and decreased cell survival	[70]
					BMDMs; peritoneal macrophages	Decreased efferocytosis and decreased cell survival	
Atherogenic *Ldlr*^*−/−*^ mice reconstituted with *Trem2*^*−/−*^ bone marrow	↓	=	↑	=	BMDMs; peritoneal macrophages	Decreased efferocytosis and decreased cell survival	[70]
4D9 (Trem2 agonist) treated atherogenic *Ldlr*^*−/−*^ mice	↑	=	↓	=	BMDMs	Increased lipid intake and increased cell survival	[70]

TREM2, triggering receptor expressed on myeloid cells 2; BMDMs, bone marrow-derived macrophages; “↑”, increased; “↓”, decreased; “=”, no significant change

## Potential of TREM2 as a therapeutic target and diagnostic biomarker for atherosclerosis

Since the mechanism of TREM2 in atherosclerosis involves multifaceted biological processes, it has broad applications for the treatment and diagnosis of atherosclerosis. Research on TREM2 as a therapeutic target for atherosclerosis is still in its early stages, yet some agonists previously used in AD models have shown promise for atherosclerosis treatment [69, 70] (Table 2). Moreover, typical applications of TREM2 in other diseases may offer valuable insights for its therapeutic use in atherosclerosis (Table 2). TREM2 has demonstrated potential as a therapeutic target in various disease models, including AD and cancer [87, 88]. Both TREM2 agonistic and blocking antibodies have been developed and utilized for this purpose, which opens up the possibility of TREM2 for the treatment of atherosclerosis. Atherosclerotic progression can be slowed down in mice with established plaques by deleting *Trem2* specifically in myeloid cells [68]. This occurred because TREM2 deficiency reduced the uptake of cholesterol by macrophages and inhibited cholesterol efflux mediated by LXR, leading to impaired proliferation and survival of foamy macrophages. However, in advanced atherosclerotic plaques, TREM2 can play a protective role by enhancing macrophage efferocytosis function, reducing the risk of plaque necrotic core formation and rupture. Recent research suggested that treating mice with the TREM2 agonist AL002a reduced necrotic core formation and improved fibrous cap development in plaques despite increasing lesion size [69]. Single-cell RNA sequencing showed that TREM2 agonism upregulated OXPHOS and collagen production gene expression in *Trem2*^*+*^*Lgals3*^*+*^*Fabp5*^*+*^ macrophages [69]. Further research is needed to understand the molecular mechanisms that drive the downstream signaling of TREM2, so that we can better understand how TREM2 can play a beneficial role at different stages of atherosclerotic plaque development.

**Table 2:** TREM2 as a therapeutic target or biomarker in atherosclerosis and other diseases

Diseases model	Category	Treatment/Biomarker	Species	Mechanism	Therapeutic effect	Ref.
Atherosclerosis	Agonist	AL002a	Mouse	Activates TREM2 signaling	Reprograms macrophage metabolism, enhances cholesterol efflux and collagen deposition and improves plaque stability	[69]
Atherosclerosis	Agonist	4D9	Mouse	1. Inhibits TREM2 shedding, increases cell surface TREM2 levels 2. Activates TREM2 signaling	Atherosclerosis treatment, enhances macrophage efferocytosis and reduces atherosclerotic plaque necrosis	[70]
Atherosclerosis	Biomarker	sTREM2	Human	sTREM2 is the product of proteolytic cleavage of the cell surface TREM2	Risk assessment, elevated sTREM2 levels are associated with disease progression.	[42, 70, 93]
Alzheimer’s disease	Agonist	AL002c	Mouse and Human	1. Activates TREM2 signaling 2. interferes with TREM2 proteolytic shedding	Enhances microglia phagocytosis and reduces inflammation	[99]
Alzheimer’s disease	Biomarker	sTREM2	Mouse and Human	sTREM2 is the product of proteolytic cleavage of the cell surface TREM2	Agonist efficacy assessment, sTREM2 reduction may indicate decreased TREM2 shedding or increased internalization.	[99]
Alzheimer’s disease	Cell replacement	Hematopoietic cell transplantation	Mouse	Replacement of dysfunctional microglia with normal or even TREM2 activity-enhanced microglia	Restores microglia function to prevent neurodegeneration	[89]
Sepsis-induced cardiomyopathy	Cell replacement	Intrapericardial administration of TREM2hi macrophages	Mouse	TREM2hi macrophages clear dysfunctional mitochondria excreted by cardiomyocytes	Improves heart damage and inflammation to save cardiac dysfunction	[21]
Myocardial infarction	Injectable gelatin hydrogel	sTREM2	Mouse	sTrem2 induces an anti-inflammatory phenotype in macrophages	Promotes improvement of cardiac structure and function after myocardial infarction	[46]
Ovarian cancer	Antibody	Fc domain-enhanced anti-TREM2 monoclonal antibody	Mouse	TREM2 expression in tumor-associated macrophages is associated with immune failure and anti-PD-1 resistance	Enhances T-cell activation and response to anti-PD-1 therapy	[100]

TREM2, triggering receptor expressed on myeloid cells 2; sTREM2, soluble TREM2; TREM2hi, high expression of TREM2

In addition to the use of antibodies, cellular replacement therapy appears to be a promising option for treating atherosclerosis. Microglial function can be restored by replacing dysfunctional *Trem2*^*−/−*^ microglia with *Trem2*^*+/+*^ microglia through systemic hematopoietic cell transplantation in the *Trem2* mutant mouse AD model [89]. Similarly, transplanting *Trem2*^*+/+*^ macrophages from healthy mice hearts into the pericardium of *Trem2*^*−/−*^ mice significantly improved septic cardiac dysfunction [21]. These findings suggest that the therapeutic effects of TREM2 can be propagated by cell replacement therapy. However, it remains to be determined how effective it is for treating atherosclerosis [46]. Notably, the protective effects of TREM2 were not necessarily dependent on intact TREM2 [90, 91]. The injection of sTREM2 in the peri-infarct region significantly improved the function and structure of the infarcted heart [46]. Ventricular injection of recombinant sTREM2 protein or adeno-associated virus mediated expression of sTREM2 has enhanced the uptake and degradation of Aβ by microglia [92]. The specific function of sTREM2 in atherosclerotic plaques is still understudied and may be a promising area for future research.

sTREM2 is a potential biomarker that can aid in the diagnosis and prognosis of atherosclerosis. Our previous research has shown that patients with coronary artery disease have elevated serum sTREM2 levels, and that sTREM2 has a higher diagnostic accuracy than traditional indicators like high-sensitivity C-reactive protein [42]. In a cohort study, serum sTREM2 can be used to predict cardiovascular death in patients with atherosclerosis [93]. Unlike other markers such as C-reactive protein, sTREM2 is produced exclusively by macrophages in plaques without interference from circulating peripheral monocytes [93]. This characteristic may make sTREM2 a more precise biomarker of atherosclerosis. However, more fundamental research is needed to clarify the biological significance of sTREM2.

## Conclusion and future directions

Studies on macrophage subpopulations in atherosclerotic plaques have highlighted the significance of TREM2hi macrophages [23, 24, 49–51, 82]. Further research is needed on the epigenetics and transcription factors of TREM2hi macrophages to better understand their regulatory mechanisms. Current studies have revealed that TREM2 is an important player in the formation, progression, and rupture of atherosclerotic plaques [67–70, 93]. These studies suggested that during early atherogenesis, TREM2 increased lesion size by promoting cholesterol uptake and macrophage survival [67, 68]. Inhibition of TREM2 may slow the lesion progression. However, in the advanced atherosclerotic plaque, TREM2 promotes plaque stability by enhancing efferocytosis to reduce necrotic core formation and reprogramming macrophage metabolism to OXPHOS [69, 70]. This is significant in decreasing the risk of major cardiovascular events. Further investigation into the molecular mechanisms is necessary, as a deeper understanding of how TREM2 regulates atherosclerosis is crucial for developing appropriate therapies.

Notably, while the role of TREM2 in reducing necrotic core formation and enhancing plaque stability is consistent across most studies, its association with increased lesion area has not been universally observed. This discrepancy may be attributed to differences in experimental models. Mechanistically, TREM2 aims to maintain macrophage viability to manage lipid overload in the arterial wall and clear necrotic debris. During this process, the increased number of surviving macrophages can lead to an expansion of the lesion area. Therefore, the enlarged lesion area might be seen as a side effect of the plaque-stabilizing function of TREM2 rather than a direct pathogenic role. However, further research is required to validate these findings. Importantly, the cellular composition of human atherosclerotic plaques is more complex, with a different proportion of macrophages compared to mice [94]. The progression rate of lesions also significantly differs between mice and humans [95]. To what extent the role of TREM2 in human plaques is influenced by these compositional and temporal differences remains unclear. A more transparent and deeper understanding of the mechanisms by which TREM2 regulates atherosclerosis is essential for the development of appropriate therapeutics.

Transcriptomic data showed that in the context of atherosclerosis, *Trem2* was predominantly expressed in various macrophage subpopulations, with the highest levels observed in foam cells [70]. Few other immune cells exhibited detectable levels of *Trem2* expression [70]. Beyond macrophages, dendritic cells also expressed lower levels of *Trem2* [70]. Some studies suggested that TREM2 may play a role in regulating homeostasis of dendritic cells and their response to pathogens, though its function in the setting of atherosclerosis remains unclear [96, 97]. Additionally, in prostate cancer, tumor-secreted APOE can bind to TREM2 on neutrophils, promoting neutrophil senescence [98]. However, the role of TREM2-expressing neutrophils in atherosclerosis has yet to be investigated.

TREM2 promotes cholesterol uptake and efflux, modulates inflammation and metabolism, and promotes cell survival, making it an attractive target for atherosclerosis research and therapy. In the future, it would be interesting to explore how to reconcile the seemingly contradictory roles of TREM2 in increasing lesion size and enhancing plaque stability in atherosclerotic lesions. Additionally, since sTREM2 serves as a marker or has therapeutic benefits in many diseases, its function in atherosclerosis and its relationship with TREM2 should be investigated. While targeting TREM2 holds promise for therapeutic interventions in atherosclerosis, several challenges need to be addressed before its clinical application. First, most current findings are based on animal models, and the translation of these results to human pathology remains uncertain due to differences in immune responses and disease progression. Additionally, the dual role of TREM2 in promoting both lesion growth and plaque stability complicates its therapeutic modulation. This stage-specific activity necessitates precise timing and regulation of therapeutic interventions. Another key challenge is that TREM2 functions in macrophages in a variety of tissues including liver, adipose tissue and brain. How to selectively target TREM2 in atherosclerotic lesions without triggering adverse effects in other tissues remains a concern for future therapeutic strategies. More research is needed to better understand how TREM2-targeted therapies can be safely and effectively applied in human patients.

## Data Availability

No datasets were generated or analysed during the current study.

## Abbreviations

ABCA1: ATP-binding cassette transporter A1
ABCG1: ATP-binding cassette transporter G1
AD: Alzheimer’s disease
ADAM: The α-secretases disintegrin and metalloproteinase
Aβ: Amyloid-β
BMDMs: Bone marrow-derived macrophages
CVD: Cardiovascular diseases
DAM: Disease-associated microglia
DAP12: DNAX activating protein 12
ER: Endoplasmic reticulum
GSK3β: Glycogen synthase kinase 3β
IL: Interleukin
ITAM: The activation motif of the immunoreceptor tyrosine
LAM: Lipid-associated macrophages
LDL: Low-density lipoproteins
LXR: Liver X receptor
mTOR: Mechanistic target of rapamycin
NAM: Nonalcoholic steatohepatitis-associated macrophages
NASH: Nonalcoholic steatohepatitis
NF-κB: Nuclear factor-kappa B
OXPHOS: Oxidative phosphorylation
oxLDL: Oxidized low-density lipoprotein
PLCγ2: Phospholipase Cγ2
PI3K: Phosphatidylinositol 3-kinase
sTREM2: Soluble TREM2
TLR: Toll-like receptor
TREM2: Triggering receptor expressed on myeloid cells 2
TREM2hi: With high expression of TREM2
VSMCs: Vascular smooth muscle cells
